# Nonsurgical Rehabilitation Perspectives for a Type I Salter-Harris Fracture With Lipohemarthrosis and Concomitant Grade II Anterior Cruciate Ligament (ACL) Tear in a Volleyball Athlete: A Case Report

**DOI:** 10.7759/cureus.59758

**Published:** 2024-05-06

**Authors:** Swapnil U Ramteke, Pratik R Jaiswal, Priya Tikhile

**Affiliations:** 1 Sports Physiotherapy, Ravi Nair Physiotherapy College, Datta Meghe Institute of Higher Education & Research, Wardha, IND; 2 Musculoskeletal Physiotherapy, Ravi Nair Physiotherapy College, Datta Meghe Institute of Higher Education & Research, Wardha, IND

**Keywords:** rehabilitation, salter-harris type 1 fracture, youth sports injuries, traumatic injuries, immature skeleton, skeletally immature athletes, anterior cruciate ligament

## Abstract

Anterior cruciate ligament (ACL) tears represent common occurrences in sports, particularly posing significant risks to young athletes. The diagnostic methods for ACL injury include magnetic resonance imaging (MRI), arthroscopy, and physical examination. Management of this injury can be done both operative and nonoperatively. Pediatric growth plate fractures are classified under the Salter-Harris classification. A 13-year-old male athlete sustained a knee injury during a volleyball match. While landing from a jump after a smash, the athlete directly landed on the ground on his right knee. After this, he reported discomfort and severe anterior knee pain. Due to immobility and pain, he was taken to the emergency unit. Upon radiographic examination, a Salter-Harris Classification Type I fracture was found, indicating epiphyseal slip and separation through the physis of the right proximal tibia medially. An MRI imaging was done to check the integrity of the ACL after the swelling had subsided post-15 days of injury. An MRI identified a bony contusion on the medial tibial plateau, extending to the physeal plate with a Grade II ACL tear. The concurrent occurrence of ACL injury and growth plate injury presents a significant concern. Hence, a referral for physical therapy rehabilitation was given. Our findings highlight the importance of prompt initiation of physical rehabilitation following such injuries. Where non-surgical rehabilitation strategies play a crucial role in managing these cases while focusing on restoring knee stability, promoting healing of the growth plate, and facilitating a safe return to sport. Tailored rehabilitation, including therapeutic exercises, neuromuscular training, and proprioceptive training, is essential for optimizing outcomes and preventing long-term complications. The case underscores the importance of a multidisciplinary approach in managing the complex knee injury of this young athlete.

## Introduction

In the past two decades, there has been a notable rise in the frequency of anterior cruciate ligament (ACL) injuries among the pediatric population, with existing estimates indicating a rate of 12 per 10,000 patients per year [1]. Moreover, there has been a notable surge in the incidence of ACL reconstruction among young athletes under the age of 20 in recent years. While this trend underscores the growing impact of ACL injuries in adolescent and teenage populations, it is important to recognize that ACL injuries affect individuals across a broad age spectrum [2]. This escalation is attributed to several factors, such as heightened engagement in sports activities from a younger age and enhanced awareness and acknowledgment of this injury. Non-contact mechanisms of ACL injury frequently entail alterations in direction, pivoting, and landing [3]. These movement patterns amplify the anterior shear force on the proximal tibia, elevating ligament stress and predisposing to lesions [4]. Following ACL surgery, individuals commonly exhibit persistent deficits in strength, proprioception, balance, and neuromuscular control, amplifying the risk of re-injury [5].

The physeal injury, during ACL reconstruction, represents a recognized complication, leading to various deformities linked with abnormal growth patterns. Damage to the physis in growing skeletons manifests gradually over time, resulting in discrepancies in leg length and angular deformities around the knee. Surgical intervention for ACL ruptures in individuals under 18 years old, particularly those who are skeletally immature, poses discrete challenges due to the existence of the growing physis [6]. While adult ACL reconstruction typically involves transphyseal sockets and tunnels, the passage of transphyseal grafts in skeletally immature athletes poses a risk of physeal damage, potentially resulting in growth disturbances. Despite efforts, orthopedic surgeons and researchers have yet to fully quantify the risks associated with angular deformities and growth disturbances arising from standard ACL reconstruction, utilizing transphyseal tunnels [7]. Traditional ACL reconstruction methods pose a risk to the distal femoral and proximal tibial physes in skeletally immature patients during tunnel drilling. Consequently, some proponents advocate for nonoperative or delayed surgical approaches to mitigate the potential for physeal injury and the associated risk of growth deformities. This includes delaying ACL reconstruction until skeletal maturity, or after a trial of nonoperative management has been attempted [8].

To mitigate the risk of growth plate disruption, the primary objective of fracture management is achieving anatomical reduction and stabilizing the fracture until complete healing is achieved [9]. Surgical reconstruction in skeletally immature patients necessitates tailored approaches to circumvent the physes or mitigate potential damage ideally. Additionally, due to differences in reconstruction methods, age, and skeletal maturity, distinct rehabilitation protocols are imperative [10]. Unlike adults, children frequently present challenges in providing a precise history of trauma mechanisms. Notably, approximately 65% of young athletes presenting with hemarthrosis are found to have an ACL rupture. Despite ongoing research, there remains a lack of consensus regarding treatment decision criteria for skeletally immature children following ACL injury [11].

Recovery for young athletes after ACL reconstruction often takes longer compared to adults due to the need for careful post-surgery measures, slower muscle strength development, and psychological considerations. Surgical procedures that prioritize the growth plates often entail restrictions on weightbearing and range of motion, adding complexity to the rehabilitation process [12]. The return-to-sport process and associated short- and long-term issues are comparable in amateur players; however, their rehabilitation may prove lengthier and more intricate compared to that of professional athletes, largely due to logistical challenges and cost considerations associated with therapy organization. Similar obstacles may also be encountered by adolescents, potentially resulting in incomplete treatment [13]. When a fracture affects the epiphyseal plate, it can lead to potential growth irregularities and structural abnormalities as the body develops. The outlook for these growth irregularities hinges on several factors, including the nature of the injury, the child's age, blood circulation to the affected area, the realignment technique used, and whether the injury occurred in a closed or open manner [14].

## Case presentation

A 13-year-old male was brought to the orthopedic department with complaints of pain, swelling, inability to bear weight on the right lower limb, and difficulty in knee bending. The patient gave a history of playing volleyball on a hard surface at his school, during which he fell on the ground, sustaining an injury to his right knee. While playing, he accidentally placed his leg over the top of the volleyball and slipped, which caused him to fall to the ground. During the fall, he landed on his right knee, with the knee at approximately 90° flexion and the ankle at plantarflexion. After the fall, pain and swelling gradually started over the right knee. Further, an orthopedician examined him, and radiological investigations were advised, which revealed epiphyseal slip and separation through the physis of the right tibia medially. He has been prescribed medications, and an above-knee slab was applied for 15 days. After 15 days, the patient visited for a follow-up, where the slab was removed, and an MRI was done, revealing a Grade II ACL tear, moderate lipohemarthrosis with suprapatellar extension, and bony contusion over medial tibial plateau extending up to physeal plate. He was advised to use a long knee brace, bed rest, and cryotherapy and was asked to come back for a follow-up after 15 days. On follow-up, he was assessed by an orthopedician, who advised him to have a hinged knee brace and was referred for physiotherapy rehabilitation. Table 1 shows the range of motion and Table 2 shows the strength assessment.

**Table 1:** Range of motion assessment.

Movement	Right				Left	
	Active		Passive		Active	Passive
	Pre-treatment	Post-treatment	Pre-treatment	Post-treatment		
Hip flexion with knee extension	0-75°	0-90°	0-80°	0-90°	0-90°	0-90°​​​​​​​
Hip extension	0-25°​​​​​​​	0-30°​​​​​​​	0-30°​​​​​​​	0-30°​​​​​​​	0-30°​​​​​​​	0-30°​​​​​​​
Hip abduction	0-40°​​​​​​​	0-45°​​​​​​​	0-45°​​​​​​​	0-50°​​​​​​​	0-50°​​​​​​​	0-55°​​​​​​​
Hip adduction	0-20°​​​​​​​	0-25°​​​​​​​	0-25°​​​​​​​	0-30°​​​​​​​	0-30°​​​​​​​	0-30°​​​​​​​
Hip internal rotation	NA	0-40°​​​​​​​	NA	0-45°​​​​​​​	0-40°​​​​​​​	0-45°​​​​​​​
Hip external rotation	NA	0-45°​​​​​​​	NA	0-50°​​​​​​​	0-45°​​​​​​​	0-50°​​​​​​​
Knee flexion	0-50°​​​​​​​	0-130°​​​​​​​	0-55°​​​​​​​	0-135°​​​​​​​	0-135°​​​​​​​	0-135°​​​​​​​
Knee extension	50-0°​​​​​​​	130-0°​​​​​​​	55-0°​​​​​​​	135-0°​​​​​​​	135-0°​​​​​​​	135-0°​​​​​​​
Ankle plantarflexion	0-50°​​​​​​​	0-50°​​​​​​​	0-55°​​​​​​​	0-55°​​​​​​​	0-50°​​​​​​​	0-55°​​​​​​​
Ankle dorsiflexion	0-20°​​​​​​​	0-20°​​​​​​​	0-20°​​​​​​​	0-20°​​​​​​​	0-20°​​​​​​​	0-20°​​​​​​​
Ankle inversion	0-30°​​​​​​​	0-30°​​​​​​​	0-35°​​​​​​​	0-35°​​​​​​​	0-30°​​​​​​​	0-35°​​​​​​​
Ankle eversion	0-15°​​​​​​​	0-15°​​​​​​​	0-20°​​​​​​​	0-20°​​​​​​​	0-15°​​​​​​​	0-20°​​​​​​​

​​​​​​​

**Table 2:** Manual muscle testing.

Muscle group	Right		Left
	Pre-treatment	Post-treatment	
Hip flexors	2/5	5/5	5/5
Hip extensors	3+/5	5/5	5/5
Hip abductor	4/5	5/5	5/5
Hip adductor	4/5	5/5	5/5
Hip internal rotators	NA	4+/5	5/5
Hip external rotators	NA	4+/5	5/5
Knee flexors	3+/5	5/5	5/5
Knee extensors	4-/5	5/5	5/5
Ankle plantar flexors	4/5	5/5	5/5
Ankle dorsiflexors	4/5	5/5	5/5
Ankle inverters	4+/5	5/5	5/5
Ankle everters	4+/5	5/5	5/5

Diagnostic imaging

**Figure 1:**
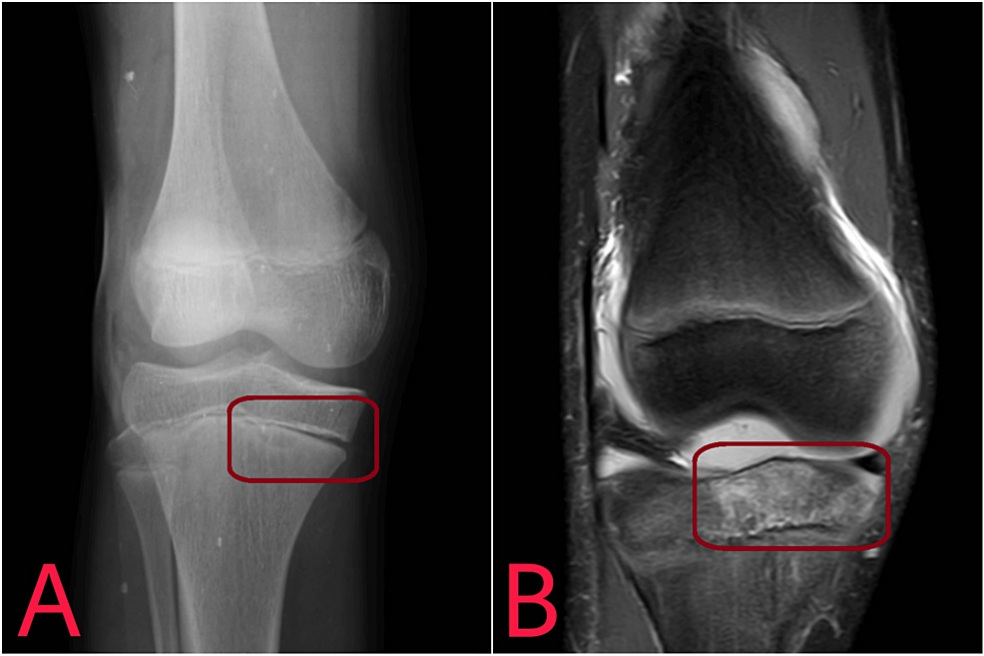
An X-ray (A) and MRI (B) showing Salter-Harris Classification Type I, i.e., epiphyseal slip and separation through the physis of proximal tibia medially.

**Figure 2:**
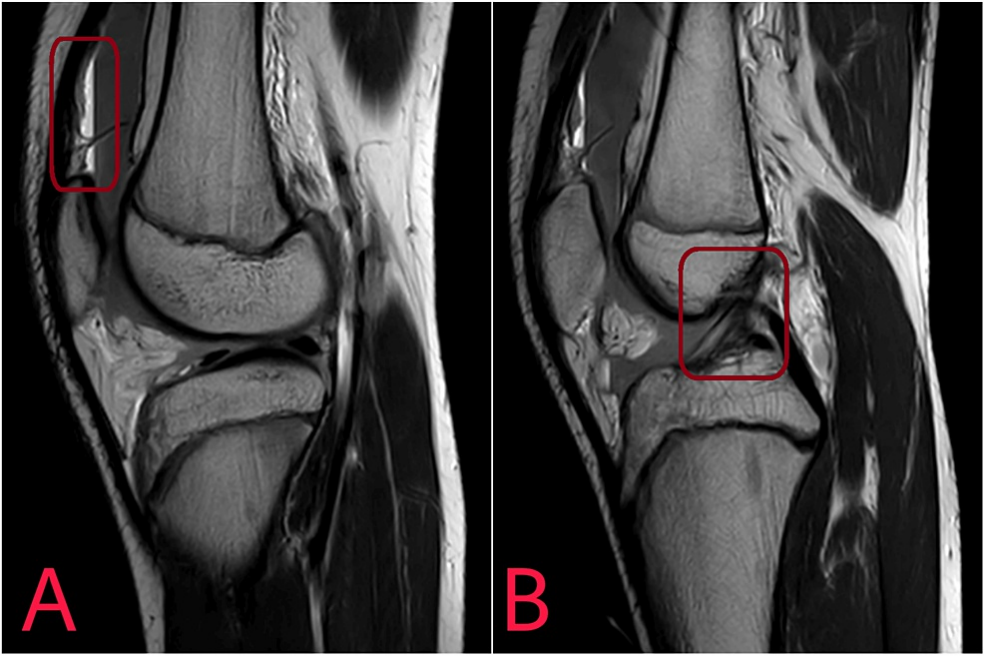
An MRI showing suprapatellar lipohemarthrosis (A) and anterior cruciate ligament tear (B).

The interpretation of radiological examination is shown in Figures 1-2.

Therapeutic intervention

The rehabilitation program was developed by taking the international classification of functioning, disability, and health into consideration. Tables 3-4 depict the same.

**Table 3:** International classification of functioning, disability, and health showing structural impairments along with source of information and clinical reasoning. ACL: anterior cruciate ligament; MRI: magnetic resonance imaging

Structural impairment	Source of information	Clinical reasoning
Partial thickness ACL tear (right side)	MRI	Trauma
Epiphyseal slip and bony contusion over medial tibial plateau	X-ray	Trauma
Atrophy of quadriceps	On observation and girth measurement	Due to trauma and reduced use of the affected limb and no weight bearing. Disuse atrophy due to immobilization.
Presence of abrasion over the anterior aspect of the proximal tibia	On observation	Trauma
Swelling and tenderness around the knee joint	On observation and palpation	Secondary to inflammation

**Table 4:** International classification of functioning, disability, and health showing functional impairments along with a source of information and clinical reasoning. MMT: manual muscle testing; ROM: range of motion

Functional impairment	Source of information	Clinical reasoning
Reduced strength of right quadriceps	MMT	Disuse atrophy
Restricted right knee flexion	ROM assessment	Adhesion formation and pain
Pain at anteromedial and anterolateral aspect of proximal tibia	Patient`s complaint and pain assessment	Due to trauma and inflammation

Tables 5-10 depict phasic rehabilitation. The therapeutic intervention focused on respective management during different phases of rehabilitation. All the precautionary measures were taken to avoid undue loads on the healing structures. The rehab guidelines strictly followed the patient's pain considerations throughout the rehabilitation program. Regular communication with the patient for any potential concerns related to treatment was maintained. During the treatment, the patient showed no adverse reactions or intolerances.

**Table 5:** Phase 1: Week 1-4 post removal of cast. The patient was immobilized in a plaster cast for a duration of two weeks; physiotherapy intervention was started post-removal of the cast. RICE: rest, ice, compression, and elevation; ROM: range of motion

Sr. No.	Goal	Intervention	Rationale
1	Educate the athlete on injury management and precautions	Explain to the athlete and parents about the nature of the injury and the expected recovery timeline. Instruct about gait training with a walker. Emphasize the importance of adherence to the rehabilitation program.	Education empowers the athlete to take an active role in their recovery and ensures compliance with the rehabilitation program.
2	Maximum protection phase	The knee brace was locked in full extension for the first week, followed by a hinge knee brace in the third week to initiate guarded healing with movement and prevent stiffness. Walker was used for non-weight bearing as per the orthopedic physician's recommendations.	Protecting the injured joint with the use of a walker and a knee brace allows for proper healing of the fracture and minimizes strain on the injured structures.
3	Reduce pain and inflammation	RICE protocol	Essential to follow in the acute stage
4	Edema control	Compression bandaging and elevation of the affected leg when lying	Elevation facilitates the reduction of edema
5	Restore normal ROM of the knee	Passive ROM exercises for maintaining joint ROM and prevention of contractures were carried out. Following this, active-assisted exercises for initiating knee flexor and extensor strength were carried out as per the patient's pain tolerance throughout the available ROM.	Early initiation of passive ROM exercises helps to prevent stiffness and maintain joint mobility. Therapist-assisted active ROM exercises can be gradually incorporated as pain allows to promote early return of active movement.
6	Prevent muscular atrophy and initiate muscle activation	Isometrics for quadriceps, hamstring, gluteal, and calf muscle activation	These exercises improve muscle activation and strength without stressing the joint, laying the foundation for future strengthening exercises. Muscle activation exercises prevent muscular atrophy.
7	Muscle reeducation	Neuromuscular electrical stimulator	It helps to maintain quadriceps muscle function and prevent muscle wasting during the initial non-weight-bearing phase.

**Table 6:** Phase 2: Week 4-8. Concentrating on restoring normal range of motion and strength as well as reducing pain and inflammation. ROM: range of motion

Sr. No.	Goal	Intervention	Rationale
1	Protection and ambulation: Introduce weight-bearing activities as tolerated	Gradual weight-bearing progressed as tolerated, transitioning from a walker to a cane.	Gradual weight-bearing progression allows for controlled stress on the healing bone and ligament, promoting continued healing and adaptation.
2	Reduce pain and inflammation to a minimal level	Continued cryotherapy as needed. Maintained pain medication as prescribed by the physician.	Continued pain management ensures the patient's comfort and adherence to the rehabilitation program.
3	Restore full knee ROM	Gradual increase in active and passive knee flexion and extension ROM. Dynamic stretches to improve flexibility.	Achieving full active ROM is crucial for optimal joint function and returning to daily activities.
4	Improve quadriceps and hamstring strength	Progressing from isometric to isotonic exercises for quadriceps, hamstrings, and calf muscles. Resistance with bands, weights, or machines was added as tolerated, and the initiation of closed kinetic chain exercises (e.g., leg press, squats).	Strengthening exercises are essential for restoring muscle function and preventing future injuries.
5	Begin proprioceptive training	Balance exercises on stable surfaces (e.g., double-leg stance) and perturbation training (gentle pushes or taps) to improve joint stability.	Proprioceptive training helps restore neuromuscular control and joint stability, reducing the risk of future injuries.
6	Enhance neuromuscular control	Proprioceptive exercises on unstable surfaces (e.g., balance board, Bosu ball).	For restoring muscle function and preventing future injuries

**Table 7:** Phase 3: Week 8-12. Focusing on strength, proprioception, and cardiovascular fitness. OKC: open kinetic chain; CKC: closed kinetic chain

Sr. No.	Goal	Intervention	Rationale
1	Improve muscular strength and endurance	Incremental resistance training with increasing intensity of loads for strengthening in OKC and CKC. Promotion of eccentric strengthening exercises. Focused single-leg and multi-joint movements.	Advanced strength training protocols aim to maximize muscular strength, power, and endurance.
2	Improve proprioception	Proprioceptive exercises on unstable surfaces (e.g., balance board, Bosu ball).	Proprioception training improves joint awareness and stability, reducing the risk of re-injury.
3	Enhance functional movement patterns specific to volleyball	Incorporating agility ladder drills, cone drills, and reaction time exercises as tolerated.	Functional movement training focuses on refining sport-specific skills and movement patterns to enhance on-court performance and reduce the risk of injury.
4	Improve cardiovascular fitness	Low-impact cardiovascular exercises (e.g., stationary cycling). Interval training to improve aerobic and anaerobic capacity.	Progressive cardiovascular conditioning prepares the athlete for the physical demands of the game.
5	Gradually reintroduce sport-specific activities	Gradually increase in intensity and duration of sport-specific activities. Monitoring signs of fatigue or pain during training sessions.	A gradual progression allows for safe reintegration into volleyball activities while minimizing the risk of re-injury and optimizing performance outcomes.

**Table 8:** Phase 4: Week 12-16. Initiating sports-specific skill training.

Sr. No.	Goal	Intervention	Rationale
1	Optimizing volleyball-specific performance abilities	Focusing on moderate intensity drills replicating game situations. Emphasizing position-specific training to address individual player needs.	Volleyball-specific performance training aims to enhance game-specific skills, conditioning, and decision-making abilities necessary for competitive play.
2	Fine-tuning technical skills and tactical awareness	Fine-tuning fundamental volleyball skills such as serving, passing, setting, hitting, and blocking. Using video analysis to provide feedback and identify areas for improvement. Targeted skill development exercises to address weaknesses.	Technical skill refinement ensures players maintain proficiency in fundamental aspects of the game, leading to improved performance outcomes.
3	Enhancing agility, speed, and power specific to volleyball movements	Integrating agility ladder drills, cone drills, and shuttle runs to improve footwork and quickness. Incorporating explosive power exercises such as jump squats and medicine ball throws. Focus on enhancing reactive strength and change-of-direction ability.	Agility, speed, and power training target key physical attributes essential for success in volleyball, including quickness, explosiveness, and agility.
4	Gradually increasing training intensity in preparation for return to competition	Gradually increasing training volume and intensity to simulate match conditions. Training with shorter rest periods to mimic the intermittent nature of volleyball. Monitoring workload and recovery to prevent overtraining and optimize performance gains.	Progressive-intensity training prepares athletes for the physical demands of competitive matches while minimizing the risk of fatigue-related injuries.

**Table 9:** Phase 5: Week 16-24. Focusing on tactical, mental, and participation skills.

Sr. No.	Goal	Intervention	Rationale
1	Achieving peak physical conditioning for return to competition	High-intensity performance training: Implementing advanced conditioning drills to improve endurance, speed, and power. Incorporating interval training with varying work-to-rest ratios to simulate match conditions. Focusing on maintaining high-intensity efforts over prolonged periods.	High-intensity performance training focuses on optimizing physical conditioning and preparing athletes for the demands of competitive volleyball matches.
2	Further improvement in volleyball-specific skills and strategies	Tactical skill development: Rotations and tactical drills. Implement role-specific training to optimize individual contributions within the team dynamic.	Tactical skill development ensures players are proficient in executing team strategies and making effective decisions during gameplay.
3	Enhance mental resilience and focus during gameplay	Mental preparation and visualization: Introducing mental skills training techniques such as imagery, positive self-talk, and goal-setting. Foster mental resilience and focus through simulated match scenarios and stress inoculation training. Encourage athletes to visualize successful performance outcomes and maintain confidence in their abilities.	Mental preparation and visualization techniques help athletes cultivate the psychological resilience and focus necessary for peak performance under pressure.
4	Prepare for full participation in competitive matches	Match simulation and scrimmages: Involving practice matches to replicate competitive conditions. Monitoring player performance and readiness for full participation.	Match simulation provides valuable opportunities for athletes to apply their skills in a competitive environment and assess readiness for full participation.

**Table 10:** Phase 6: Week 24-36. Preparing the athlete for return to sports.

Sr. No.	Goal	Intervention	Rationale
1	Achieve full physical readiness for return to competitive play	Full-intensity training and conditioning: Implementing high-intensity training sessions mirroring match conditions, including full-court drills and simulated gameplay. Emphasizing maintaining peak physical conditioning and minimizing fatigue-related performance decrements. Gradually, tapering training volume and intensity to ensure the athlete is prepared for competition.	Full-intensity training and conditioning ensure athletes are physically prepared to meet the demands of competitive volleyball matches without risking injury or performance decrements.
2	Optimal performance under game conditions	Game-specific skill refinement: Focusing on volleyball-specific skills and strategies through game-like simulations and situational drills. Addressing any remaining technical or tactical deficiencies identified during training sessions.	Game-specific skill refinement focuses on fine-tuning technical and tactical aspects of performance to optimize on-court success.
3	Monitoring for any signs of residual impairment or risk of re-injury	Psychological readiness and mental preparation: Conducting pre-competition mental preparation sessions to enhance focus, confidence, and resilience. Addressing any performance anxiety or psychological barriers through supportive interventions and coping strategies.	Psychological readiness and mental preparation help athletes cultivate the mindset and resilience necessary for peak performance under pressure.
4	Providing ongoing support and guidance for athletes' transition back to competition	Return-to-competition assessment: Conducting comprehensive physical assessments to evaluate readiness for full participation. Monitoring for any signs of residual impairment or risk factors for re-injury. Collaborating with medical and coaching staff to make informed decisions regarding return to competitive play.	Return-to-competition assessment provides a systematic approach to evaluating athlete readiness and minimizing the risk of re-injury during the transition back to competitive play.

Table 11 depicts the outcome measures.

**Table 11:** Outcome measures.

Outcome measures	Pre-treatment	Post-treatment
Lysholm Knee Score	11%	95%
The International Knee Documentation Committee	24%	95%
Knee Injury and Osteoarthritis Outcome Score	27%	94%

**Figure 3:**
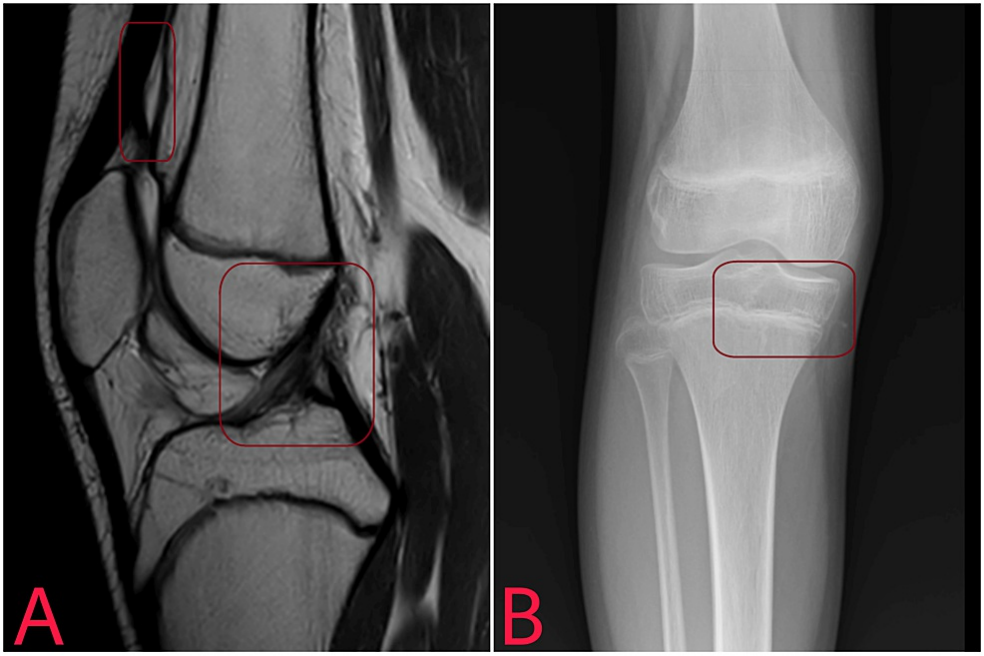
An MRI (A) showing a healed suprapatellar lipohemarthrosis and anterior cruciate ligament. An X-ray (B) showing a reunited epiphyseal plate of the proximal tibia.

The interpretation of radiological examination post-rehabilitation is shown in Figure 3.

## Discussion

Advanced surgical repair techniques are promising as potential options for skeletally immature patients, but their efficacy necessitates extensive long-term evaluation. It is crucial to recognize non-surgical treatments or ligament repair techniques based solely on outdated methodologies. Continued scrutiny of repair techniques is warranted, considering recent and forthcoming studies in this field [15]. In their systematic review, Filbay et al. concluded that early surgical reconstruction does not yield substantial enhancements in patient-reported outcomes among the young adult population compared to non-surgical or delayed surgical approaches [16]. In a prospective study by Moksnes et al., 46 skeletally immature children (aged 12 years and younger) were subject to a non-operative treatment regimen comprising a structured physical therapy program and bracing, aimed at restoring functional knee stability. Upon final follow-up, 22% of patients had opted for surgical reconstruction due to symptomatic ACL deficiency. Notably, 52% of patients had successfully resumed their previous level of sports activity at the one-year mark, with 50% maintaining this level at the two-year evaluation. The authors concluded that non-operative treatment might be suitable for some ACL-injured youth, although others might necessitate a reduction in activity level. However, achieving a reduced activity level in such an active patient cohort is not always practical or desirable [17].

Greenberg et al. noted that certain pediatric patients may exhibit substantial muscle deficits for over a year post-ACL reconstruction and could potentially benefit from an extended rehabilitation period [18]. Quadriceps muscle weakness has been linked with ACL injury and has proven challenging to recover without a well-structured rehabilitation program post-injury [19]. Effective management of ACL injury hinges on comprehensive and systematic rehabilitation exercises, irrespective of whether the patient opts for surgical reconstruction or conservative treatment. While rehabilitation principles in children are largely extrapolated from adult clinical experience and research, their applicability to pediatric populations remains uncertain. Further prospective studies are warranted to evaluate rehabilitation protocols and return-to-sport criteria tailored to young athletes, considering both physical and psychosocial distinctions between children and adults [20].

Acute traumatic injuries in young athletes often mirror those seen in adult athletes; however, the affected structures may differ significantly. Young athletes, with immature skeletons, possess open growth plates and more fragile bones, rendering them susceptible to fractures and cartilage injuries rather than muscular and ligamentous damage typically observed in adults. Fortunately, most fractures heal without complications when promptly identified and managed. Growth plate disturbances can accompany various types of Salter-Harris fractures, imposing thorough evaluation for potential fractures before diagnosing a soft tissue injury in youth athletes.

According to our knowledge, this is the first case reporting the simultaneous injury of epiphyseal plate slip, along with a Grade II ACL tear, in a skeletally amateur athlete. As nonoperative rehabilitation was preferred in this particular case, the time taken to complete the rehab was more than usual cases. However, any chances of secondary complications were avoided due to nonoperative rehabilitation. The rehabilitation protocol employed in this case can be modified and applied accordingly in the future.

Precaution

Ideally, the rehabilitation of ACL injury includes a plyometric training program, but considering the epiphyseal fracture, plyometric should be done with precautions.

Limitation

It's important to note that the conservative approach taken in rehabilitating the skeletally immature athlete required a gentler and more cautious approach than typically applied to adults. Failure to adhere to a proper rehabilitation protocol could lead to early-onset osteoarthritis of the knee as a potential long-term complication. Additionally, premature return to sports without adequate rehabilitation may enhance the risk of further injury. Early initiation of physical therapy is imperative in the rehabilitation process for injured athletes, facilitating optimal recovery.

## Conclusions

The rehabilitation trajectory for this specific case differs from traditional ACL rehabilitation protocols for adults due to distinct forms of injury and age. The current rehabilitation timeline relies heavily on known ACL healing patterns in adults and the expertise of surgeons. Future research is imperative to establish precise timelines and benchmarks for strength recovery, evaluate the suitability of ACL functional performance measures for skeletally immature individuals, and define normal performance standards for young groups. Moreover, comprehensive long-term studies are essential to gauge the proportion of patients achieving successful outcomes over time.
